# Bovine Lactoferrin Prevents Influenza A Virus Infection by Interfering with the Fusogenic Function of Viral Hemagglutinin

**DOI:** 10.3390/v11010051

**Published:** 2019-01-11

**Authors:** Fabiana Superti, Mariangela Agamennone, Agostina Pietrantoni, Maria Grazia Ammendolia

**Affiliations:** 1 National Centre for Innovative Technologies in Public Health, National Institute of Health, Viale Regina Elena 299, 00161 Rome, Italy; agostina.pietrantoni@iss.it (A.P.); maria.ammendolia@iss.it (M.G.A.); 2 Department of Pharmacy, University “G. d’Annunzio”, Via dei Vestini 31, 66100 Chieti, Italy; m.agamennone@unich.it; 3 Core Facilities, National Institute of Health, Viale Regina Elena 299, 00161 Rome, Italy

**Keywords:** influenza virus, fusion peptide, lactoferrin, antiviral agents

## Abstract

Bovine lactoferrin (bLf) is an iron-binding glycoprotein folded in two symmetric globular lobes (N- and C-lobes) with potent antimicrobial and immunomodulatory activities. Recently, we have shown that bLf, and in particular its C-lobe, interacts with influenza A virus hemagglutinin and prevents infection by different H1 and H3 viral subtypes. Influenza virus hemagglutinin (HA), and in particular its highly conserved fusion peptide involved in the low-pH-mediated fusion process, plays a significant role in the early steps of viral infection and represents an attractive target for the development of anti-influenza drugs. In the present research, we further investigated the influence of low pH on the interactions between bLf and influenza A H1N1 virus by different techniques, such as enzyme-linked immunosorbent assay, electron microscopy, hemolysis inhibition assay, and time course assay. Our results demonstrate that lactoferrin interaction with influenza hemagglutinin at low pH induces alterations that stabilize the conformation of the hemagglutinin, resulting in the inhibition of the fusion peptide activity. Taken together, our data allowed to better characterize the HA-specific inhibiting activity of bLf and to confirm HA as a good target for drug development.

## 1. Introduction

**Figure 1:**
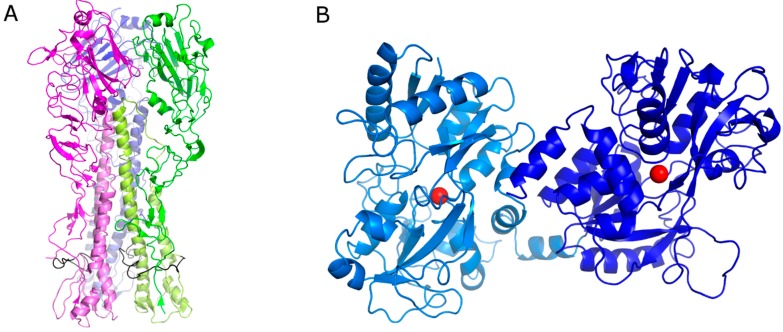
(**A**) Cartoon representation of the hemagglutinin (HA) trimeric protein. Each monomer has a different color. The HA1 chain has been depicted with a dark color and HA2 with a pale color. The fusion peptide is represented in black. (**B**) Cartoon representation of bovine lactoferrin (bLf). The N-lobe is represented in pale blue and the C-lobe in blue. The iron ions are depicted as red spheres.

Influenza A virus respiratory infections are a serious cause of morbidity and mortality worldwide, particularly among aged and high-risk people. Two treatment strategies are currently recommended in the case of infection: vaccination and antiviral drug treatment. Vaccination represents the main approach for the prevention and control of seasonal influenza; nevertheless, vaccine effectiveness can vary depending upon a number of factors, including the genetic relationship among the viruses used for the vaccine and circulating strains. The commercially available FDA-approved anti-influenza drugs hit two viral targets: the matrix protein 2 ion channel (amantadine and rimantadine) and neuraminidase (oseltamivir, peramivir, laninamivir, and zanamivir). All the approved anti-influenza drugs have several limitations, such as their potential side effects, the limited efficacy, and the emergence of new viral strains refractory to conventional treatments [1,2,3,4,5]. Therefore, there is an urgent need to develop novel compounds targeting different viral proteins to address the issue of drug resistance [6]. In particular, the ideal therapeutic target should be a viral protein critical for infectivity, such as hemagglutinin (HA). HA, a glycoprotein expressed on the viral envelope along with neuraminidase (NA), is pivotal for the interactions between the influenza virus and sialic acid side chains of receptors on the host cell surface [7] and is also required for the final step of viral entry into susceptible cells, i.e., the pH-dependent fusion between the viral envelope and the cellular endosomal membrane [8]. HA also plays a significant role in host immune responses by harboring the major antigenic sites responsible for the production of neutralizing antibodies. Mature HA is a homotrimer composed by three identical subunits, with a mushroom-like shape (Figure 1A). Each HA subunit consists of two disulfide-linked polypeptide chains, HA_1_ and HA_2_. HA_1_, constituting most of the globular head of HA, contains the sialic acid binding site [9,10,11] and undergoes recurring mutations to evade antibody protection (antigenic drift). HA_2_ occupies the stem region and contains most of the membrane fusion machinery of HA. This would explain why inhibiting HA can result in two outcomes: (i) prevention of the interaction between viral surface proteins and cell surface receptors; (ii) blocking of viral envelope fusion with the host cell membrane and thereby the release of viral nucleoproteins into the cytoplasm.

Lactoferrin (Lf) is an 80-kDa multifunctional cationic glycoprotein belonging to the transferrin family and possessing a variety of biological functions, such as an influence on iron homeostasis, immunomodulation, and inhibitory activity towards different pathogens [12,13,14]. Bovine lactoferrin (bLf) has been recognized as a potent inhibitor of different viruses and has been often reported to exhibit higher antiviral activity than human lactoferrin (hLf) [15,16]. BLf, similarly to hLf, is folded in two symmetrical lobes (N- and C-lobes) with high sequence homology, possibly resulting from an ancestral gene duplication [17,18] (Figure 1B).

We have recently observed that lactoferrin from bovine milk is able to prevent both influenza virus infection and hemagglutination [19,20,21]. Moreover, we demonstrated that lactoferrin specifically binds the HA_2_ subunit of HA [21]. In particular, we ascertained that the bLf C-lobe is responsible for these activities.

In the present study, we attempted to further investigate the interaction between bovine lactoferrin and HA in order to better characterize the antiviral activity mechanism of this protein. Obtained results show that lactoferrin is able to block HA_2_ functions. As a matter of fact, we demonstrated that lactoferrin is able to prevent the early steps of influenza virus infection and that its interaction with viral particles is improved at low pH, when the hydrophobic NH_2_-terminal amino acid residues of HA_2_ are exposed. Consistent with this result, lactoferrin (and its C-lobe) also blocked virus-induced hemolysis of erythrocytes in a dose-dependent manner.

## 2. Materials and Methods

### 2.1. Cells

Madin–Darby canine kidney (MDCK) cells were grown in minimum essential medium (MEM, Invitrogen, Carlsbad, CA, USA) and used for virus propagation as already described by us [19,20].

### 2.2. Virus

The H1N1 influenza A virus strain A/RomaISS/2/08 H1N1, kindly provided by Dr. Isabella Donatelli (National Institute of Health, Rome), was prepared by infecting monolayers with 1 plaque-forming unit (p.f.u.)/cell, as already described by us [19,20]. Briefly, after 90 min incubation at 35 °C, cells were layered with culture medium supplemented with 4% bovine serum albumin (BSA, fraction V, Gibco; Paisley, UK) and 0.5 µg of N-tosyl-L-phenylalanine chloromethyl ketone-treated trypsin (Sigma Chemical Co., St. Louis, MO, USA) and incubated at the same temperature until extensive cytopathic effect (c.p.e.) was observed. Then, after at least three freezing and thawing cycles, cell debris was pelleted (10 min at 3000 rpm) and supernatants were stored at −80 °C. Viral HA activity was revealed by hemagglutination test, whereas viral infectious particles were quantified by plaque assay [22].

Virus purification was carried out as already described by us and reported in the European patent number EP 2,780,365 B1 [23]. Briefly, after differential centrifugation, clarified infected supernatants were layered onto a discontinuous sucrose gradient (from 0% to 60%) and centrifuged at 85,000× *g* for 2 h. Purified viral particles were collected from the 20/40% sucrose interface and stored at −80 °C [23].

### 2.3. Lactoferrin and Ammonium Chloride

Lactoferrin from bovine milk (bLf) was obtained from Morinaga Milk Industries (Zama City, Japan). Endotoxin deprivation, purity checking, protein concentration, and iron saturation rate were assayed as previously described [19,24,25]. Detoxified bLf and ammonium chloride (NH_4_Cl, Sigma Chemical Co., St. Louis, MO, USA) were dissolved as stock solutions (0.25 mM and 400 mM, respectively) in pyrogen-free phosphate buffered saline (PBS, pH 7.4). Cytotoxicity was evaluated according to a previously reported method [19].

### 2.4. Hydrolysis of bLf and Characterization of Its C-lobe

The procedures employed to obtain, purify, and characterize the C-lobe have been carried out as previously reported by us [24,26]. Briefly, after bLf enzymatic digestion, the C-lobe was purified by reversed-phase high-performance liquid chromatography and analyzed by sodium dodecyl sulphate–polyacrylamide gel electrophoresis and mass spectrometry to check its identity and purity.

### 2.5. Effect of Lactoferrin on Influenza Virus Infection: Time Course Assay

The effect of 12.5 μM bLf on the different steps of influenza virus infection was tested in a time-of-addition assay. For these experiments, infection was synchronized by incubating the virus (10 plaque forming units per cell) with the cells at 4 °C for 1 h (attachment step). After this time, cells were washed twice with medium to remove unbound viral particles and incubated for 6 h at 37 °C to allow virus internalization. The inhibiting effect of bLf was assessed by three different experimental procedures: (i) infected cells were treated with bLf for the entire time of infection (6 h at 37 °C); (ii) infected cells were treated with bLf for different periods of time; (iii) infected cells were incubated for well-defined lengths of times before the addition of bLf. Influenza virus antigen synthesis was measured by indirect immunofluorescence. 

### 2.6. Indirect Immunofluorescence Staining

MDCK cells, grown and infected on coverslips, were washed in PBS and fixed with ice-cold acetone for 5 min. Cells were then incubated with monoclonal IgG raised against purified influenza virus type A strain H1N1 (Santa Cruz Biotechnology, cat. sc-52025) for 45 min at 37 °C. After washing in PBS, viral antigen synthesis was estimated by utilizing anti-mouse IgG (whole molecule)–FITC antibody produced in goat (Sigma-Aldrich cat. F0257), and cell nuclear staining was achieved using 0.1 µg/mL Hoechst 33,342 (10 min at 37 °C). Data for immunofluorescence were collected on an Olympus BX 53 microscope and captured with a digital CCD camera Tucsen USB 2.0 H series. ISCapture software program was utilized to acquire, manage, and process the images. Hoechst 33,342 was utilized to count the entire cell population and to discriminate between infected and mock-infected cells. The ratio between total cells and infected cells was utilized to evaluate the percentage of infected cells.

### 2.7. Enzyme-Linked Immunosorbent Assay (ELISA)

To determine bLf binding to viral particles pretreated at an appropriate pH, an ELISA was carried out. The A/RomaISS/2/08 virus was treated with 0.1 M Tris, 1 M NaCl, 0.05 M Na–EDTA (TNE buffer) at different pH (pH 7.4, 6.0, 5.0, and 4.0). After incubation at 37 °C for 15 min, the reaction was neutralized with NaOH and the different virus samples were used for the binding assay. BLf (12.5 µM/well, corresponding to 0.1 mg/well) dissolved in carbonate buffer (0.05 M) was used for coating flat-bottomed 96-well plates (Nalge Europe Ltd., Neerijse, Belgium) which were incubated overnight at 4 °C. Then, plates were put at 37 °C, and after blocking with BSA (fraction V, Gibco; Paisley, UK; 10% in PBS) for 2 h, were incubated with 50 µL viral particles and pretreated at different pH for 1 h. After washing, chicken anti-influenza A antibodies (Abcam plc, Cambridge, UK) diluted in 1% BSA (in PBS) were added and the plates were incubated for 1 h at 37 °C. Then, plates were washed again and horseradish peroxidase (HRP)-conjugated rabbit anti-chicken IgG antibody (Sigma Chemical Co.; St. Louis, MO, USA) was added for 1 h. After washing with PBS-T (10 mM phosphate buffer pH 7.4, 150 mM NaCl, 0.05% Tween 20), the peroxidase substrate o-phenylendiamine (OPD) dihydrochloride was added. The developing of the color reaction was stopped by adding 50 μL of 3.0 N HCl. Viral particles pretreated at the indicated pH were used as standards. The optical density was read on a multilabel plate reader (PerkinElmer, Monza, Italy) at 490 nm. The virus binding to lactoferrin was calculated as follows: the absorbance value of each virus sample, pretreated at different pH, was considered as 100%; after subtraction of nonspecific binding, the virus binding to lactoferrin was obtained as a percentage relative to each virus control.

### 2.8. Transmission Electron Microscopy (TEM)

For TEM visualization, purified viral particles were pretreated at different pH (from 7.4 to 4.0), and after pH neutralization, 20 μL of each sample was absorbed onto Formvar carbon-coated, 400-mesh copper grids. Negative staining was carried out by utilizing 2% phosphotungstic acid (PTA) (pH 7.0) for 30 s, and samples were visualized by an EM 208 FEI transmission electron microscope at 80 kV.

### 2.9. Hemolysis Inhibition Assay

A modification of the hemolysis inhibition assay described by Bodian and coworkers [27] was carried out. Briefly, solutions of purified viral particles (100 μL; about 6 μg of protein) were incubated with 100 μL solutions containing different amount of bLf or bLf C-lobe in PBS for 1 h at 37 °C. To this mixture, a solution of turkey erythrocytes (200 μL, 2.0% in PBS) was added and again incubated at 37 °C for 10 min. Turkey red blood cells (RBCs) were pelleted by centrifugation at 1600 rpm for 8 min and the pellet was resuspended in 450 μL of low-pH PBS buffer (pH 5.0 ± 0.05) containing the corresponding concentrations of bLf or bLf C-lobe. After 25 min of incubation at 37 °C, the reaction was neutralized to pH 7.0 ± 0.2 by the addition of 1 N NaOH. Cell debris and unlyzed cells were pelleted down through centrifugation at 2000 rpm for 8 min. Supernatant optical density was read on a multilabel plate reader (PerkinElmer Italia, Monza) at 540 nm. Background values were derived from mock-infected samples that underwent identical treatment.

Percentage of protection was calculated by the equation (A540 protein–virus − A540 virus)/(A540 PBS − A540 virus) × 100%.

### 2.10. Statistical Analysis

Data from three independent experiments were reported as the mean ± standard deviation (SD). Statistical analysis was carried out using one-way analysis of variance (ANOVA). The critical value for the statistical significance was *p* ≤ 0.05.

## 3. Results

### 3.1. Lactoferrin Inhibits the Early Phases of Influenza Virus Infection

**Figure 2:**
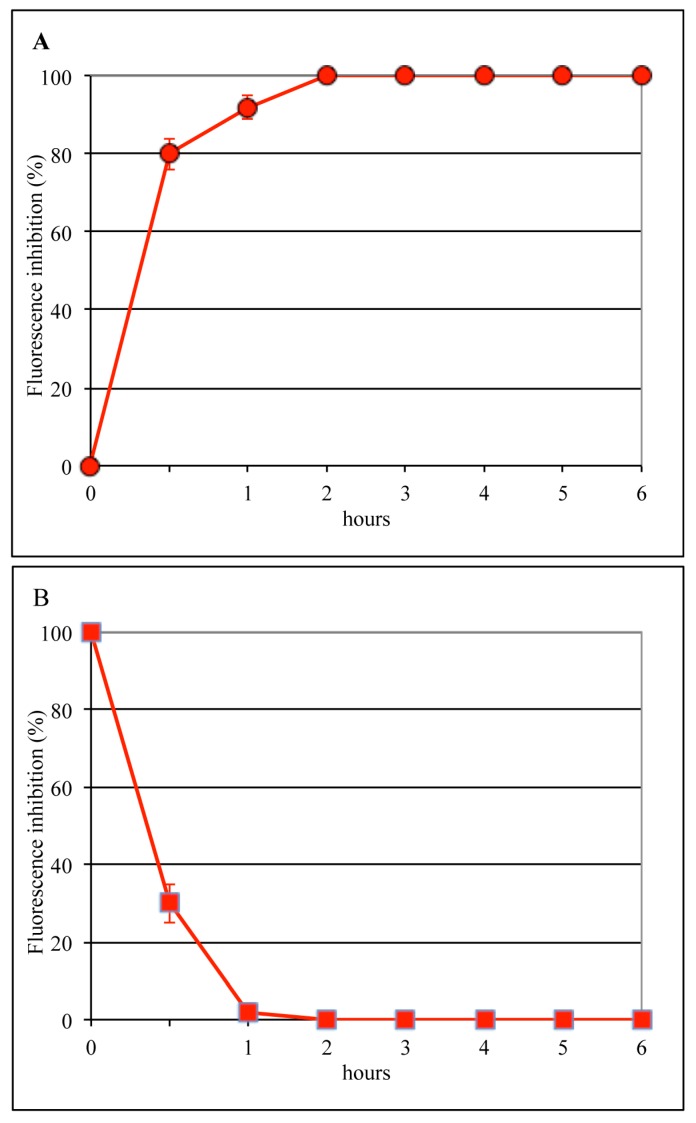
Dependence of 12.5 µM bLf time of addition on infection by the A/RomaISS/2/08 H1N1 strain. BLf was incubated with the cells directly after the virus-binding step and left for different periods of time (**A**), or, after virus adsorption, infected cells were kept at 37 °C for various periods of time before bLf addition (**B**). Six hours after viral infection, the synthesis of viral antigens was assessed by indirect immunofluorescence.

To evaluate the infection step affected by bLf, experiments were carried out in which MDCK cells were incubated with the virus for 1 h at 4 °C and then bLf (12.5 μM) was added for different lengths of time (Figure 2A) or at different times (Figure 2B) after the viral attachment step (time course assay). It was found that bLf totally prevented viral antigen synthesis when present during the entire cycle of infection, whereas no inhibition was observed when it was added 60 min after viral binding. Moreover, 70% inhibition of viral antigen synthesis was obtained when the protein was present during the first 30 min of infection. Results from these experiments showed that bLf antiviral effect was exerted between 30 and 60 min from the temperature shift to 37 °C; this time frame corresponds to the early steps of influenza virus infection, such as internalization or membrane fusion (uncoating).

**Figure 3:**
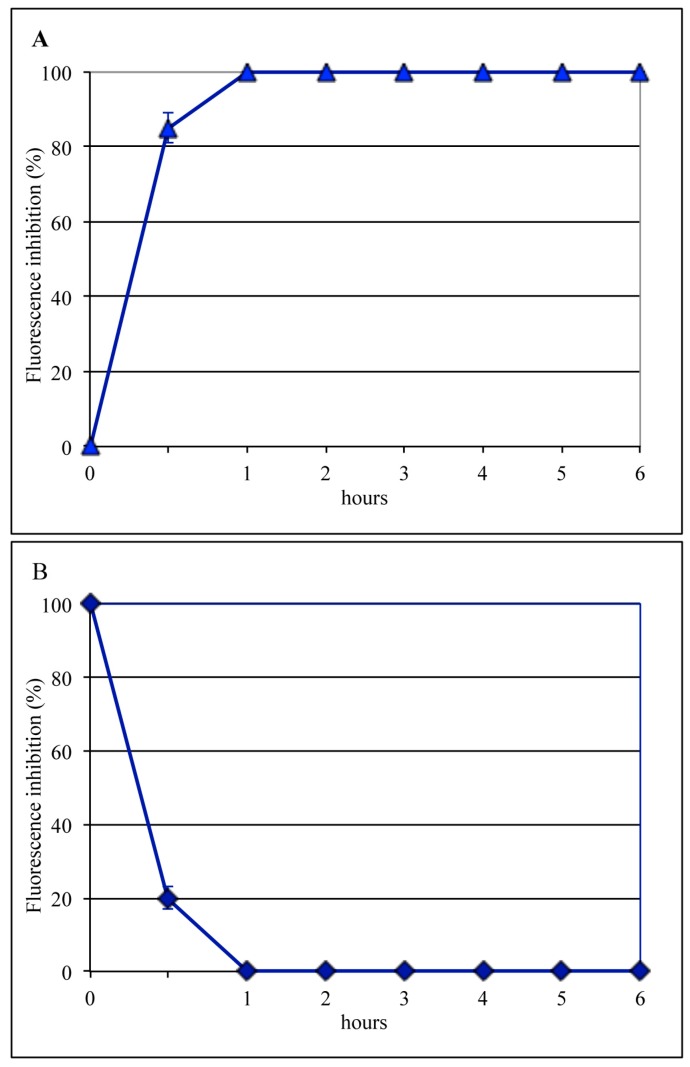
Addition-time effect of 20 mM NH_4_Cl on influenza virus infection. (**A**) NH_4_Cl was added to the medium immediately after the attachment step, followed by incubation for different lengths of time. (**B**) After virus attachment, cells were incubated for various periods of time before the addition of NH_4_Cl. Six hours after viral infection, the synthesis of viral antigens was assessed by indirect immunofluorescence.

To identify the post-adsorption stage sensitive to bLf, the same time-of-addition assay was performed using NH_4_Cl, which buffers the pH of acidic cellular compartments and is able to prevent infection by viruses that require a low pH for uncoating. Results of these control experiments (Figure 3) showed that NH_4_Cl, like bLf, totally prevented viral antigen synthesis when present during the entire cycle of infection, whereas when incubated for 1 h after viral binding, the inhibition was 100% for NH_4_Cl and 92% for bLf. Both compounds were ineffective when added 1 h after viral internalization (Figure 2 and Figure 3). Taken together, these results suggest that both compounds could act on the same infection step, i.e., uncoating.

### 3.2. Direct Lactoferrin Binding Assay at Low pH

The influenza virus enters the host cell through endocytosis, followed by the fusion of the virus with endosomal membranes, viral uncoating, and export of the viral genome into the cell. The mechanism of membrane fusion, triggered by low pH, has been widely characterized: the endosomal low pH induces conformational changes in the HA protein, leading to insertion of a hydrophobic ‘fusion peptide’ into the target cell membrane.

**Figure 4:**
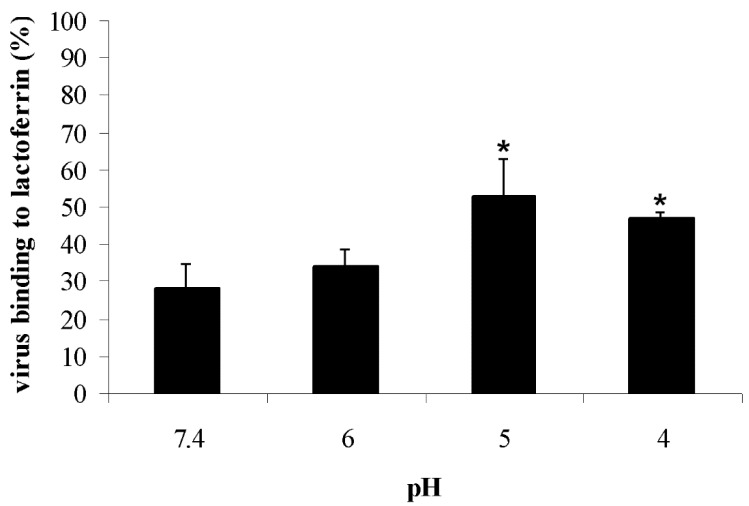
Influence of pH on influenza virus binding to bLf by ELISA. Virus samples incubated with buffers at different pH (from 7.4 to 4.0) were absorbed to bLf-coated plastic surfaces. The interaction between the different influenza virus samples and bLf was analyzed by an enzyme-linked immunosorbent assay, and results were expressed as the percentage of virus binding to bLf. Error bars represent the mean of three independent experiments. * *p* < 0.05.

The pH of endocytic compartments varies from about 6.0 (early endosomes) to 5.0 (late endosomes) and 4.0 (when the late endosome fuses with the lysosome). To verify whether the pH-dependent structural rearrangement of HA could influence the interaction between lactoferrin and viral particles, experiments were carried out in which bLf binding to purified viral preparations treated with different acidic buffers (pH 7.4, 6.0, 5.0, and 4.0) was quantified by an ELISA. Results obtained are reported in Figure 4. BLf was found to bind to the virus with the highest affinity at acidic pH (52.9 ± 9.7% and 47.2 ± 1.4% at pH 5.0 and 4.0, respectively). The protein binding strength decreased at pH 6.0 (34.1 ± 4.1%) and was lower at pH 7.4 (28.2 ± 6.35%).

### 3.3. Transmission Electron Microscopy of Untreated and Acid-Treated Virus Particles

**Figure 5:**
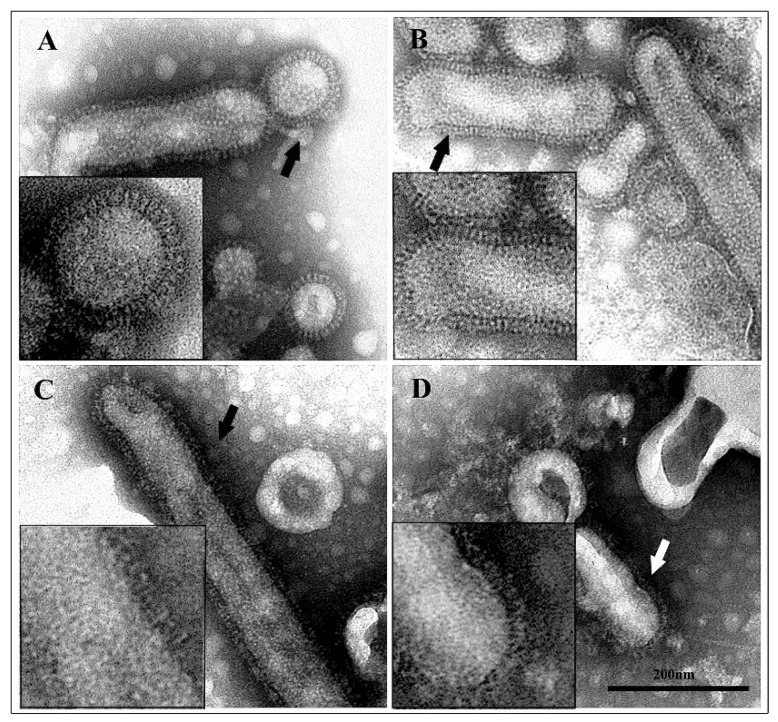
Negative staining of influenza virus particles. At pH 7.4, virions show well-ordered surface spikes (**A**); at pH 6.0, spikes are still well-defined (**B**); after exposure to pH 5.0, virions show a disordered surface (**C**); at pH 4.0, the spikes appear very discontinuous (**D**). Inserts show greater detail.

Purified viral preparations treated with different acidic buffers (pH 7.4, 6.0, 5.0, and 4.0) were observed by transmission electron microscopy after negative staining with phosphotungstic acid. Untreated and acid-treated virus particles markedly differed in their morphology (Figure 5). At neutral pH, the HA spikes were well-ordered, rectangular structures projecting from the viral membrane. Viral particles treated at pH 6.0 showed still distinct and well-defined spikes on the virions. After incubation at pH 5.0, the well-ordered appearance was lost and individual spikes were difficult to discern. This pattern was more evident after treatment at pH 4.0, which induced HA spikes to appear very disordered and barely distinguishable. The loss of well-defined spike structure observed at low pH reflects the irreversible conformational change of HA [28].

### 3.4. Inhibitory Effect of Lactoferrin and Lactoferrin C-lobe on Hemolysis

**Figure 6:**
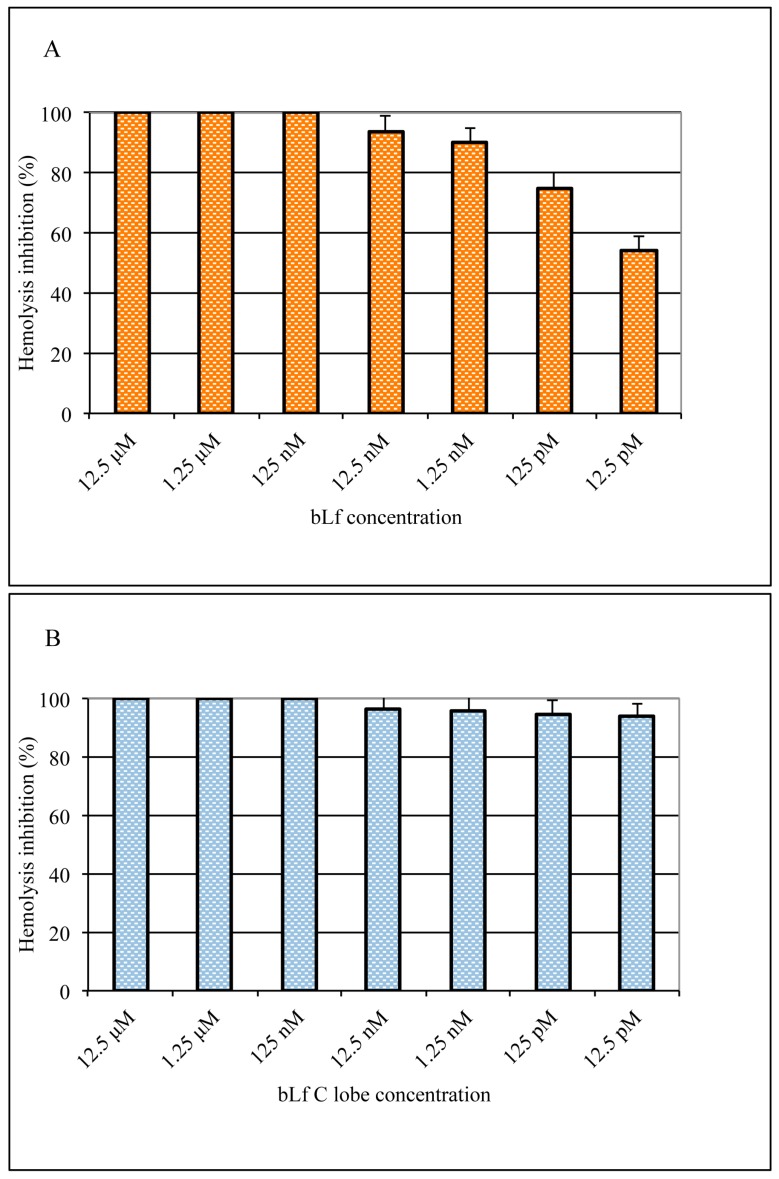
Hemolysis inhibition (%) by different concentrations of bLf (**A**) and bLf C-lobe (**B**). Total (100%) hemolysis was defined by the amount of hemoglobin released in the absence of bLf (virus alone, OD 1.335).

To confirm that bLf targets the viral fusion activity, we used a hemolysis inhibition test. A suspension of viral particles and turkey erythrocytes was incubated for a short time at low pH to allow alterations in the hemagglutinin, inducing the production of ‘pores’ on the erythrocyte membrane through which hemoglobin content can be released. Results obtained showed that both bLf (Figure 6A) and bLf C-lobe (Figure 6B) completely inhibited hemolysis at the concentration of 125 nM when added to virus–erythrocyte suspension before acidification. However, at lower concentrations, A/RomaISS/2/08 H1N1 was more sensitive to bLf C-lobe compared to the entire protein.

## 4. Discussion

**Figure 7:**
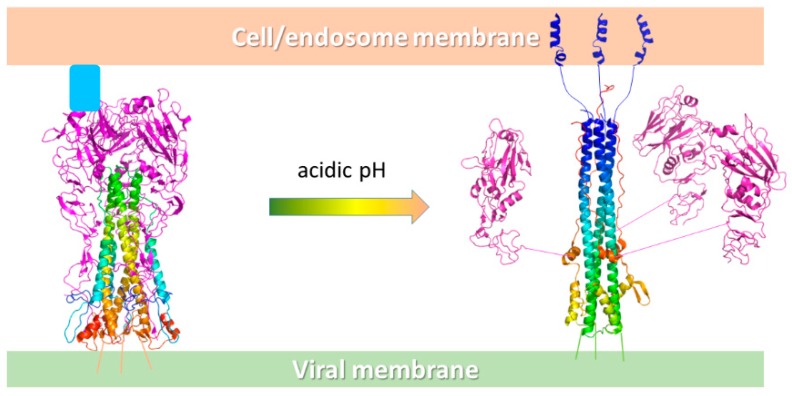
Schematic representation of the conformational change of HA at acidic pH. On the left, the HA exposed on the viral surface binds the host cell receptor sialic acids (pale blue box) on the HA_1_ binding sites (purple cartoon), while the HA_2_ trimer forms the central stem (rainbow cartoon). At acidic pH, the HA_2_ portion undergoes a relevant conformational rearrangement with the fusion peptide (blue helices) that moves 100 Å away from the original position and inserts into the endosomal membrane. The process ends with the fusion of the viral and host membranes and the release of the viral material into the infected cell.

The major influenza virus envelope glycoproteins are hemagglutinin (HA) and neuraminidase (NA) [29]. Both HA and NA functions are mediated by sugar side chains on cell surface receptors; in fact, while HA is responsible for the binding of the virus to target cells and the entry of the viral genome into the cytoplasm by binding cell surface sialic acids, NA is involved in the release of progeny virus from infected cells, by cleaving the bond between the sialic acid of cell surface receptors and HA. Thus, both these proteins represent chief targets for antiviral drugs. NA is the target of compounds called neuraminidase inhibitors, such as oseltamivir and zanamivir. NA inhibitors are, at present, the primary treatment against influenza virus infection, and along with M2 ion channel inhibitors (amantadine and rimantadine), represent the only weapon against influenza. However, they can cause side effects, have limited efficacy if administered late in infection, and their widespread use is likely to induce the emergence of resistant viral strains [30,31,32]. Consequently, HA represents a very promising target for developing new drugs against influenza virus infection. HA is homotrimeric and each monomer is composed of two disulfide-linked polypeptides, identified as HA_1_ and HA_2_. The HA_1_ segments are responsible for hemagglutinin binding to the host cell. This step is followed by viral endocytosis and by an irreversible structural HA modification within the acidic endosomal pH (∼5.0–5.5). This irreversible conformational adjustment concerns the release of the hydrophobic fusion peptide, mediating the fusion between the viral and endosomal membranes and the release of the viral ribonucleoprotein into the cytoplasm (Figure 7) [10].

It is important to note that since the fusion peptide is essential for the release of the viral genome into the cell and is the only conserved sequence in both influenza A and B hemagglutinin, compounds blocking the activity of this peptide are very attractive in the fight against ‘the flu’. In this framework, we have previously demonstrated that bLf recognizes the HA_2_ subunit of influenza virus HA, which is involved in pH-induced conformational changes during influenza virus entry into susceptible cells [21]. In the present work, we attempted to better characterize the action of bLf on the influenza virus, and in particular, we investigated the infection step inhibited by the protein as well as the influence of pH in bLf antiviral activity.

It is well known that the influenza virus enters susceptible cells by receptor-mediated endocytosis [33]. After internalization into early endosomes (pH ∼6), the virus is transferred along the endocytic pathway to acidic late endosomes, where low pH (∼5) exposure triggers HA-catalyzed fusion between the viral and endosomal membranes, resulting in viral ribonucleoprotein release into the cytoplasm [33,34,35]. Immunofluorescence studies showed influenza virus colocalization with early endosomal markers about 10 min post-infection and with late endosomal markers approximately 40 min after infection [36]. Moreover, by using conformation-specific hemagglutinin antibodies, it has been observed that HA does not attain a fusion-competent form until the virus has trafficked outside the early endosomes [36].

Results of time-of-addition experiments, showing that the inhibitory activity of bLf was totally abolished when the protein was added one hour after viral binding, demonstrated that bLf blocks influenza virus replication at an early phase of the infection process, probably the viral late endosomal membrane fusion (uncoating). One possibility is that at the low pH of the late endosomes, when the hydrophobic NH_2_-terminal amino acid residues of HA_2_ are exposed outside, bLf may efficiently interact with HA. As a matter of fact, bLf is able to inhibit viral infection by more than 90% when present in the first hour of infection. To confirm this hypothesis, control experiments were carried out to test the ability of NH_4_Cl to prevent viral infection. In fact, it is well-known that NH_4_Cl, raising the endosomal pH [37], is able to prevent viral uncoating [38,39]. As expected, NH_4_Cl treatment totally prevented viral infection. Interestingly, we demonstrated, by comparing the effect of NH_4_Cl and bLf in a time-of-addition assay, that the temporal kinetics of the inhibitory activity of both compounds were quite similar. These results suggest that NH_4_Cl and bLf, although acting with different mechanisms, operate at the same stage, namely the prevention of viral uncoating.

It has been reported by many authors that lactoferrin, like the influenza virus, enters the cells by endocytosis [40,41,42] and is found in endosome vesicles [43]. As the acidic environment of the endosome (pH ~5.0) triggers conformational changes in HA that expose the fusion peptide, and as already demonstrated that bLf recognizes the fusion domain of HA [21], we hypothesized that bLf could block viral infection through a specific interaction with the fusion peptide at low pH. To confirm this hypothesis, we analyzed the capability of plastic-adsorbed bLf to capture acid-pretreated viral particles by an ELISA. Results of these experiments suggest that the low-pH-induced conformational change of HA resulted in a better interaction between bLf and influenza virions. Interestingly, bLf was able to bind to the virus with the highest affinity at pH 5.0, when the fusion peptide is completely exposed.

Then, to verify if the spike array was actually affected by the pH, the same viral preparations were then observed by transmission electron microscopy. Results of ultrastructural examination demonstrated that, as already reported in literature [28,44], at low pH, the viral spikes become conspicuously disorganized. The loss of well-defined spike structure observed at pH 5.0 reflects the irreversible conformational change in HA [28]. So, it can be assumed that at low pH, when the well-defined spike structure is lost, bLf may sit in a region and act as a wedge, directly blocking the movement of either the fusion peptide or another region of the HA molecule.

Finally, the role of bLf in the prevention of HA-mediated fusion was investigated by a hemolysis assay. In these experiments, the activity of the entire protein was compared to that of its C-lobe. Results obtained suggest that bLf binds in the stem region of the HA trimer and inhibits HA-mediated fusion in a dose-dependent manner. In addition, our results, as already demonstrated by us [21], confirm that the anti-influenza activity of bLf is exclusively expressed by its C-lobe.

Taken together, the results described in this study about the anti-influenza activity of bLf have led to a better characterization of this HA-specific inhibitor, confirming HA as a good target for drug development.

## Patent

Part of the results showed in this manuscript are reported in the European patent number EP 2,780,365 B1, published 22 February 2017.
